# Performance Evaluation of the MatMaCorp COVID-19 2SF Assay for the Detection of SARS-CoV-2 from Nasopharyngeal Swabs

**DOI:** 10.1128/spectrum.00083-21

**Published:** 2021-08-11

**Authors:** Arryn Craney, Dustin Petrik, Ashley Sukhu, Yuqing Qiu, Sabrina Racine-Brzostek, Hanna Rennert, Heather Piscatelli, Govardhan Rathnaiah, Alyssa Hangman, Michael Carrie, Melissa Cushing

**Affiliations:** a Department of Pathology and Laboratory Medicine, New York Presbyterian Hospital–Weill Cornell Medicine, New York, New York, USA; b MatMaCorp, Lincoln, Nebraska, USA; c Department of Population Health Sciences, Weill Cornell Medicine, New York, New York, USA; Memorial Sloan Kettering Cancer Center

**Keywords:** COVID-19, MatMaCorp, RT-PCR, SARS-CoV-2, Solas 8, point of care

## Abstract

The coronavirus disease 2019 (COVID-19) pandemic has taken an unprecedented toll on clinical diagnostic testing, and the need for PCR-based testing remains to be met. Nucleic acid amplification testing (NAAT) is the recommended method for the diagnosis of severe acute respiratory syndrome coronavirus 2 (SARS-CoV-2) due to the inherent advantages in sensitivity and specificity. In this study, we evaluated the performance of the MatMaCorp COVID-19 2SF test, a reverse transcription-PCR (RT-PCR) assay for the qualitative detection of SARS-CoV-2 from nasopharyngeal (NP) swabs, run on the Solas 8 instrument (MatMaCorp, Lincoln, NE). The Solas 8 device is portable, and the kit is a lab-in-a-box design which provides reagents in a shelf-stable lyophilized powder format. A total of 78 remnant clinical specimens were used to evaluate the COVID-19 2SF test. Sixty-two clinical specimens originally tested by the Xpert Xpress SARS-CoV-2 assay (Cepheid, Inc., Sunnyvale, CA) were used to evaluate the clinical accuracy of the COVID-19 2SF test. The negative percent agreement (NPA) was 100% (95% confidence interval [CI], 83.9% to 100%), and the positive percent agreement (PPA) was 85.4% (95% CI, 70.8% to 94.4%). Sixteen remnant specimens positive for other common respiratory pathogens (FilmArray respiratory panel 2.0; BioFire, Salt Lake City, UT) were assayed on the Solas 8 device to evaluate specificity. No cross-reactivity with other respiratory pathogens was identified. The unique lab-in-a-box design and shelf-stable reagents of the MatMaCorp COVID-19 2SF test offer laboratories a rapid option for a diagnostic NAAT for SARS-CoV-2 that can help meet diagnostic needs.

**IMPORTANCE** The demand for molecular testing for COVID-19 remains to be met. This study of the MatMaCorp Solas 8 device and COVID-19 test provides the first evaluation of this platform.

## INTRODUCTION

In late December 2019, a cluster of pneumonia of an unknown etiology was reported from Wuhan, China (1). The agent was identified as a novel coronavirus, severe acute respiratory syndrome coronavirus 2 (SARS-CoV-2), and the disease was named coronavirus disease 2019 (COVID-19). This new viral pathogen has spread globally and as of March 2021 has been responsible for ∼500,000 deaths in the United States and ∼2.5 million deaths worldwide (https://covid19.who.int/; accessed 21 February 2021). Diagnostic testing is key to controlling this new agent of infectious disease.

Nucleic acid amplification testing (NAAT) is one of the recommended diagnostic methodologies for SARS-CoV-2 (https://www.cdc.gov/coronavirus/2019-ncov/testing/diagnostic-testing.html). This demand has led to global shortages in commercial NAAT testing kits for SARS-CoV-2 and the resources for laboratory-developed tests such as nucleic acid extraction kits. Timely reporting of SARS-CoV-2 results is essential for successful epidemiological efforts to control SARS-CoV-2 spread. Centralized testing can delay result reporting, and access to the specialized equipment required for molecular testing in an ambulatory setting may be limited. Thus, meeting the demand for diagnostic testing has been challenging, and more testing resources are urgently needed to this day (2).

In December 2020, the MatMaCorp COVID-19 2SF test, performed on the Solas 8 device, received emergency use authorization (EUA) from the U.S. Food and Drug Administration for the qualitative detection of SARS-CoV-2 in upper respiratory specimens (nasopharyngeal [NP] swabs, midturbinate swabs, and anterior nasal swabs) from individuals suspected by their health care provider of having COVID-19. The COVID-19 2SF test is a molecular *in vitro* diagnostic test that utilizes a combined reverse transcription-PCR (RT-PCR) and isothermal nucleic acid amplification reaction, targeting the RNA-dependent RNA polymerase (RdRp) coding region of the Orf1ab polyprotein of SARS-CoV-2 (3). The COVID-19 2SF assay is performed on the Solas 8 instrument (MatMaCorp, Lincoln, NE), in two processes. First, samples are processed by chemical lysis and heat inactivation. This is followed by reverse transcription-PCR (RT-PCR) and isothermal amplification for detection of SARS-CoV-2. Each Solas 8 device can process up to 6 clinical specimens and 2 control samples per run. The run time is estimated at ∼2 h, including 20 min of hands-on time.

Here, we evaluated the clinical performance of the MatMaCorp COVID-19 2SF test using remnant clinical specimens.

## RESULTS

The clinical performance of the MatMaCorp COVID-19 2SF assay was evaluated using a total of 78 clinical specimens. A total of 62 specimens were assayed for SARS-CoV-2 using the Xpert Xpress SARS-CoV-2 assay. Of these, 41 specimens were positive for SARS-CoV-2, with threshold cycle (*C_T_*) values ranging from 17.6 to 34.4 (mean, 26.6; standard deviation [SD], 5.2) based on the SARS-CoV-2-specific gene (N2 gene) amplified in the Xpert Xpress SARS-CoV-2 assay (Table 1). After initial testing, 36 samples were concordant positive, 21 were concordant negative, and 6 showed discordant results (Table 2). The negative percent agreement (NPA) and positive percent agreement (PPA) were 100% (95% confidence interval [CI], 83.9% to 100%) and 85.4% (95% CI, 70.8% to 94.4%), respectively (Table 2). The six discordant results were repeated on the reference assay (Table 3). A single discordant result failed to repeat with the reference method, and the remaining five were successfully detected. These 5 remaining false negatives by the COVID-19 2SF assay had higher *C_T_* values (≥30) according to the reference assay.

**TABLE 1:** SARS-CoV-2 *C_T_* value^*a*^ range of remnant specimens used in this study

SARS-CoV-2 detection result	No. of specimens
Positive, *C_T_*	
>30^*b*^	12
20–30	23
<20	6
Negative	21
Total	62

a *C_T_* values of the N2 gene from the Xpert Xpress SARS-CoV-2 assay.

b *C_T_* values ranged from 30.4 to 33.5.

**TABLE 2:** Summary of the clinical evaluation of the COVID-19 2SF assay^*a*^

COVID-19 2SF assay result	Standard-of-care assay result (no.)		
	Positive	Negative	Total
Positive	35	0	35
Negative	6	21	27
Total	41	21	62

a Positive percent agreement (PPA), 85.4% (95% confidence interval [CI], 70.8 to 94.4%). Negative percent agreement (NPA), 100% (95% CI, 83.9 to 100%).

**TABLE 3:** Summary of six discordant results and investigation testing

Specimen no.	Xpert Xpress SARS-CoV-2 assay *C_T_* value^*a*^ (initial testing)	MatMaCorp COVID-19 2SF (observed result)	Xpert Xpress SARS-CoV-2 result (repeat testing)	Resolution (yes/no)
1	27	Not detected	Not detected	Yes
2	30.9	Not detected	Detected	No
3	31.8	Not detected	Detected	No
4	32.2	Not detected	Detected	No
5	32.3	Not detected	Detected	No
6	33	Not detected	Detected	No

a *C_T_* values of N2 gene from the Xpert Xpress SARS-CoV-2 assay.

An additional 16 clinical specimens positive for respiratory pathogens other than SARS-CoV-2 were included. These specimens were tested on the FilmArray respiratory pathogen panel prior to the introduction of SARS-CoV-2 (Table 4). All 16 specimens tested negative by the COVID-19 2SF assay, in agreement with the *in silico* predictions of specificity performed by MatMaCorp (3).

**TABLE 4:** Nasopharyngeal specimens (*n* = 16) with alternative respiratory pathogens

Specimen no.	Pathogen(s) present
1	Coronavirus HKU1
2	Coronavirus HKU1 and respiratory syncytial virus
3	Coronavirus OC43
4	Coronavirus NL63
5	Coronavirus 229E, adenovirus, and rhinovirus/enterovirus
6	Adenovirus and respiratory syncytial virus
7	Human metapneumovirus
8	Human rhinovirus/enterovirus
9	Parainfluenza virus 1
10	Respiratory syncytial virus
11	Influenzae A (2009)
12	Influenzae A (H3)
13	Influenza B
14	Mycoplasma pneumoniae
15	Chlamydia pneumoniae
16	Bordetella pertussis

## DISCUSSION

Access to timely testing for the detection of SARS-CoV-2 is critical for the curbing the COVID-19 pandemic (2). The MatMaCorp COVID-19 2SF assay provides a new option with EUA for diagnosis by NAAT for SARS-CoV-2 that offers a simple design for low-resource settings interested in surveillance testing or for Clinical Laboratory Improvement Amendments (CLIA)-certified clinical laboratories unable to meet their SARS-CoV-2 testing needs.

The reagents, including controls for the MatMaCorp COVID-19 2SF assay, are shelf stable, do not require refrigeration, and are rehydrated for use with just molecular-grade water. The Solas 8 device performs both cell lysis and amplification/detection of SARS-CoV-2, negating a need for additional molecular equipment. Importantly, the MatMaCorp COVID-19 2SF extraction design requires only a lysis buffer (included in the test kit) and a heat supply (provided by the Solas 8 device), avoiding supply chain issues for most extraction kits. The COVID-19 2SF assay is performed on the MatMaCorp Solas 8 device, with six clinical specimens analyzed per run along with a positive and negative control. The total run time is ∼2 h, including about 20 min of hands-on time. High-viral-load samples will be called positive as early as 15 min into the amplification reaction, taking these positive calls to a total time of ∼1 h and 30 min. The Solas 8 can be accessed by a local area network (LAN) or Wi-Fi network, or by directly connecting to the instrument through its access point (AP) router feature.

The COVID-19 2SF test performed well, with a NPA of 100% (95% CI, 83.9% to 100%) and a PPA of 85.4% (95% CI, 70.8% to 94.4%). Six discordant results were observed during the evaluation of the COVID-19 2SF test. Upon further investigation, the discordant specimens were retested by the reference method (Xpert Xpress SARS-CoV-2 assay). All discordant results were false negatives by the COVID-19 2SF assay (Table 3). All six patients were symptomatic at time of specimen collection. Due to the limited availability of additional assays, an independent third NAAT method could not be used to assess inaccuracies in the original testing method. For 5 of the 6 discordant specimens, repeat testing with the original comparator assay detected SARS-CoV-2 in the clinical specimen. For a single discordant result, SARS-CoV-2 was not detected by the comparator assay on repeat testing, suggesting that the stability of this specimen may have been compromised. While care was taken to store specimens quickly and testing occurred within the manufacturer’s specifications, it is unknown if this specimen was subject to additional freeze-thaws during storage or if there were any delays prior to freezing the specimen (during transport or initial refrigeration) that may have compromised the specimen. All remaining five false negatives had high *C_T_* values according to the original comparator assay (*C_T_* ≥ 30; Xpert Xpress SARS-CoV-2 assay), suggesting a low viral burden. The limit of detection (LOD) reported by each of the two manufacturers supports this difference in assay sensitivity, with the Xpert Xpress SARS-CoV-2 assay reported limit of detection at 250 copies/ml and that of the COVID-19 2SF assay at 2,000 copies/ml (3, 4). However, the 2-min heat inactivation process negates the need for a more complex extraction process that would likely improve the assay’s sensitivity. Importantly, the performance of the MatMaCorp assay was within the range of those for other diagnostic testing platforms for SARS-CoV-2 (5–9). For example, one study reported the LOD of the ePlex SARS-CoV-2 assay (GenMark Dx, Carlsbad, CA), which uses proprietary eSensor technology, at 1,000 copies/ml, and that of the isothermal amplification assay ID Now COVID-19 (Abbott Diagnostics Scarborough, Inc., Scarborough, ME) at 20,000 copies/ml (7).

This study has a few limitations. The study evaluated the clinical performance of the MatMaCorp COVID-19 2SF assay using frozen remnant clinical specimens, which thus may alter the assay performance. Unfortunately, due to the limited available testing for SARS-CoV-2, a third assay for SARS-CoV-2 could not be used to assess discordant results. As well, only a small sample size was evaluated, and the LOD was not evaluated. However, full performance characteristics are available in the package insert (3). A limitation of the MatMaCorp COVID-19 2SF test itself is that the RNA input volume for the reaction is only a few microliters of the clinical specimens. This limited input is due to the MatMaCorp COVID-19 2SF assay using a heat-inactivated lysate instead of purified nucleic acid. The use of unpurified lysates limits the sensitivity of the assay because a large volume of the original specimen cannot be concentrated through the nucleic acid extraction process, and substances from the clinical specimen that interfere with the amplification and detection process of NAAT are not removed. Notably, while the RNA input volume limits the sensitivity of the MatMaCorp COVID-19 2SF assay, the assay does not require additional resources for nucleic acid extraction. This may be of benefit if resources are limited.

In this study, we evaluated the clinical performance of the MatMaCorp COVID-19 2SF assay for the detection of SARS-CoV-2 from NP specimens. We tested a total of 78 clinical specimens, of which 6 were discordant, all in low viral-load-specimens. The MatMaCorp SARS-CoV-2 assay offers an alternative platform to meet diagnostic needs for SARS-CoV-2.

## MATERIALS AND METHODS

### Specimen collection and handling and reference method testing.

A total of 78 nasopharyngeal (NP) specimens were collected by flocked swab in universal/viral transport medium (UTM/VTM) by qualified health care professionals. All remnant specimens were frozen within 8 h of clinical testing. A total of 62 specimens were collected from patients meeting the clinical or epidemiological criteria for SARS-CoV-2 testing. These samples were originally assayed with the Xpert Xpress SARS-CoV-2 assay (Cepheid, Inc.), which served as the reference method for comparison to the MatMaCorp COVID-19 2SF test (MatMaCorp, Lincoln, NE) (Table 1). A total of 41 of the samples were positive, with *C_T_* values spanning the diagnostic range, and 21 samples were negative for SARS-CoV-2 detection by this reference method. See Table 1 for a specimen breakdown. Specimens were analyzed by the Xpert Xpress SARS-CoV-2 assay according to manufacturer’s instructions. To explore the specificity of the MatMaCorp COVID-19 2SF test, an additional 16 specimens were included that were positive for other respiratory pathogens by the FilmArray respiratory pathogen 2.0 panel (BioFire). Due to the lack of availability of remnant clinical specimens positive to evaluation for respiratory pathogens other than SARS-CoV-2 during the study period, these additional specimens were collected from patients prior to the introduction of the novel infectious agent, SARS-CoV-2. See Table 4 for a specimen breakdown. Specimens were analyzed according to manufacturer’s instructions.

### The MatMaCorp COVID-19 2SF assay and Solas 8 platform.

**FIG 1:**
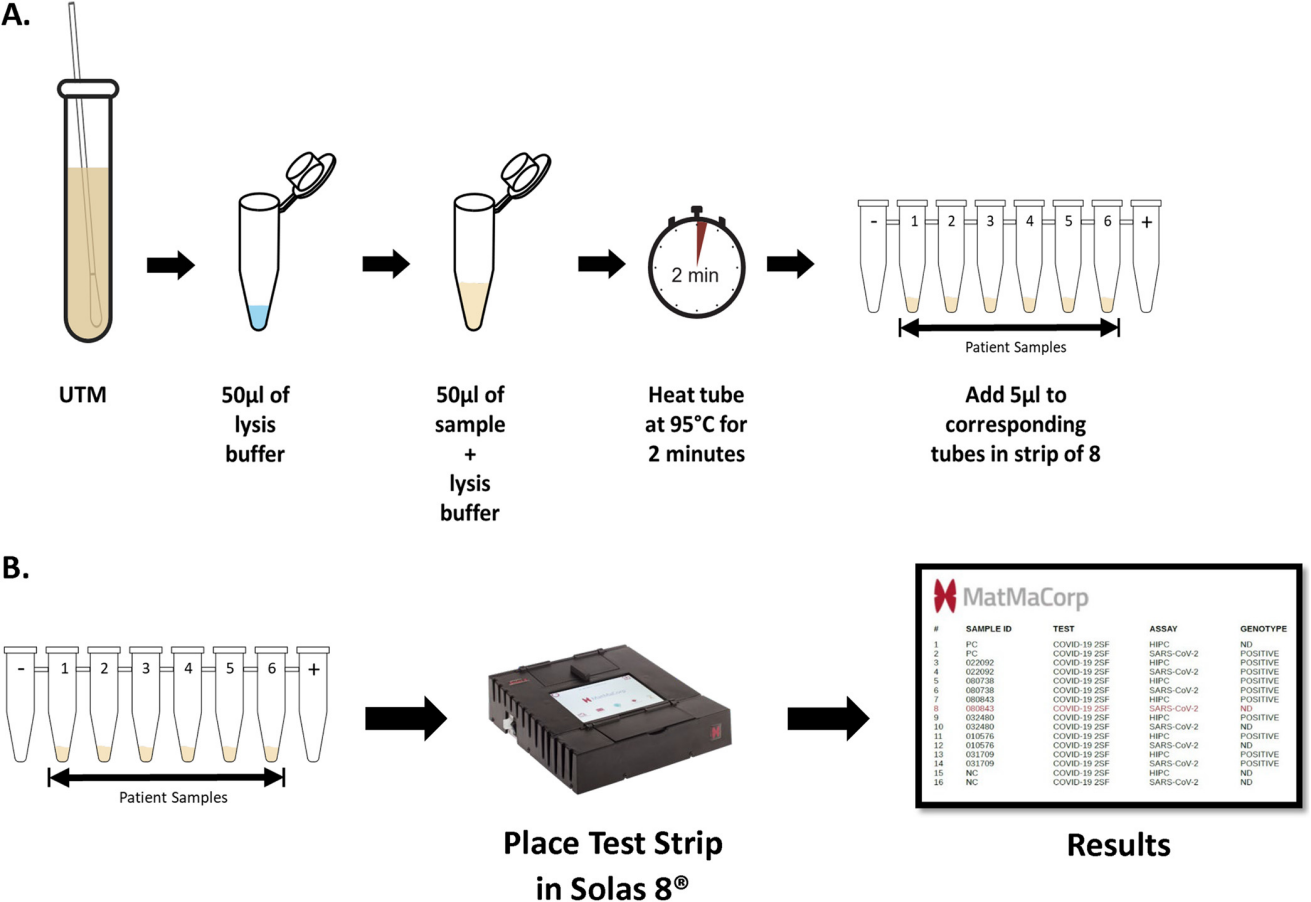
MatMaCorp COVID-19 2SF test workflow. (A) Equal volumes of nasopharyngeal (NP) swab specimen in universal/viral transport medium (UTM/VTM) are combined with CNL (lysis) buffer in a 1.5-ml lid-locking tube, then heated for 2 min at 95°C on the Solas 8 platform. Only 5 μl of the sample lysate is used in the reaction mixture. The reaction is performed in 8-tube PCR strips. Up to 6 patient samples can be analyzed per run. Tube 1 is a negative control (NC), and tube 8 is a positive control (PC); both are provided in the kit. (B) Reaction tubes are loaded into the Solas 8 instrument for reverse transcription, probe binding, and isothermal amplification. Fluorescent detection is automatically translated into calls of “positive” or “not detected” (ND) for each sample and target. Calls are displayed in real time on the screen of the Solas 8 instrument, and a portable document format (PDF) file with final results is generated at the end of the run.

The MatMaCorp COVID-19 2SF test utilizes a combined RT-PCR and isothermal nucleic acid amplification technology for the qualitative detection of the Orf1ab polyprotein gene of SARS-CoV-2 on the MatMaCorp Solas 8 instrument. Each Solas 8 instrument can run 6 clinical specimens and one positive and one negative control per run. An internal processing control is included in each reaction targeting a human gene. The assay comprises the following two procedures: (i) sample processing and (ii) amplification and detection. Sample processing occurs by mixing a 1:1 (vol/vol) ratio of the NP specimen and lysis buffer (included in the kit) and heating the sample in the big block of the Solas 8 for 2 min at 95°C. Amplification and detection of SARS-CoV-2 consists of 2 steps in which lyophilized reagents are rehydrated and added to the assay sequentially. Step 1 performs cDNA synthesis and an initial PCR amplification using a padlock probe specific to each target. Step 2 allows for the detection of the targets using fluorescently labeled probes and isothermal rolling circle amplification (RCA). These reactions occur in the small block of the Solas 8, with a total reaction time estimated at ∼2 h. Calls of “positive” or “not detected” (ND) are made in real time by the instrument, without user analysis, and results are populated into a report file in portable document format (PDF) (Fig. 1).

### Clinical evaluation of the MatMaCorp COVID-19 2SF assay.

Frozen remnant clinical specimens were tested according to the manufacturer’s instructions (3). Briefly, 50 μl of specimen was used as input for sample extraction. Samples were extracted by adding the specimen to 50 μl of CNL (lysis) solution and heating for 2 min at 95°C in the Solas 8 instrument. For detection, 5 μl of the heated lysate is used as input for the COVID-19 2SF assay. Discordant results were resolved by repeat testing of the clinical specimen by the reference method, the Cepheid Xpert Xpress SARS-CoV-2 assay (4).

### Statistical analysis.

The positive percent agreement (PPA) and negative percent agreement (NPA) for the 62 specimens tested by the Xpert Xpress SARS-CoV-2 assay were calculated using R (10) and the *epiR* statistical package (11).

### Institutional review board.

This study was performed under a Weill Cornell Medicine Institutional Review Board-approved protocol (approval no. 20-03021671).
